# Engagement in Youth Athletes as a Positive Experience in Sport: Implications of Gender, Age, and Competitive Level

**DOI:** 10.3390/ejihpe14060106

**Published:** 2024-06-04

**Authors:** María Julia Raimundi, Ignacio Celsi, Mauro Pérez-Gaido, Vanina Schmidt, Isabel Castillo, Octavio Alvarez

**Affiliations:** 1 National Scientific and Technical Research Council (CONICET), Buenos Aires C1425FQB, Argentina; juliaraimundi@mdp.edu.ar (M.J.R.); ignaciocelsi@conicet.gov.ar (I.C.); mpgaido@untref.edu.ar (M.P.-G.); vschmidt@psi.uba.ar (V.S.); 2 Institute of Basic, Applied and Technological Psychology (IPSIBAT), National University of Mar del Plata, Mar del Plata B7603ETK, Argentina; 3 Faculty of Psychology and Human Relations, Interamerican Open University (UAI), Buenos Aires C1147AAU, Argentina; 4 Research Institute, Faculty of Psychology, University of Buenos Aires, Buenos Aires C1052AAA, Argentina; 5 Department of Health and Social Security, National University of Tres de Febrero, Buenos Aires C1062AB0, Argentina; 6 Department of Social Psychology, Faculty of Psychology and Speech Therapy, University of Valencia, 46010 Valencia, Spain; octavio.alvarez@uv.es

**Keywords:** engagement, athlete, youth sport, positive youth development, health

## Abstract

The aim of this study was to examine athlete engagement and its relationships with indicators of the quality of the athlete’s sport experience, exploring potential differences according to gender, age, and competitive level. Furthermore, this study validated the Athlete Engagement Questionnaire (AEQ) in young athletes and confirmed its factor structure. A total of 1188 athletes (43.90% girls) from Argentina participated in the study, with a mean age of 15.92 (*SD* = 2.50). The participants completed the AEQ along with other measures of athletes’ quality of experience, such as motivation, enjoyment, and burnout. This study confirmed the multidimensional nature of engagement, showing positive associations with high-quality athlete experiences and revalidating the inverse relationship with burnout. Moreover, the study found differences in engagement dimensions (i.e., confidence, vigor, dedication, and enthusiasm) based on the interplay of gender, age, and competitive level. In general, male athletes, younger athletes, and those with a higher competitive level showed more engagement and interactions between these sociodemographic variables. The Argentinian version of AEQ exhibited optimal fit and reliability and good indexes of measurement invariance across gender, age, and competitive level. These findings validate the AEQ as a reliable tool for evaluating sport engagement among adolescents in Argentina. Engagement constitutes an indicator of an optimal experience linked to positive youth development through sports participation.

## 1. Introduction

Sport represents an excellent setting for health and positive youth development. Thus, it can foster social, physical, and psychological well-being, providing a beneficial environment for optimal development and the acquisition of a healthy lifestyle, especially during adolescence [1]. However, despite these benefits, there are circumstances in which young athletes may face negative and adverse outcomes, such as stress, pressure, and burnout [2]. Several studies have suggested that healthy behaviors, well-being, and athletic performance are associated with athletes’ experiences, such as self-determined motivation and enjoyment, rather than the sport itself [3,4].

Engagement is known as one of the ideal experiences that athletes can develop through their practice. It is considered a persistent experience with core dimensions that involves a long period of sports participation rather than a state [5]. This optimal experience, as a high-order construct, is defined as a persistent and positive cognitive-affective process characterized by the belief in one’s own capacity to achieve a certain level of performance and goals (confidence), the desire to invest effort and time in the pursuit of important objectives (dedication), the presence of physical, mental, and emotional energy or vitality (vigor), and the feeling of enjoyment in the sporting activity (enthusiasm) [5,6].

Engagement has been identified as the opposite of burnout syndrome [7]. In the context of sports, burnout is the most common experience of distress [8]. It is characterized by exhaustion, devaluation, and a reduced sense of accomplishment [9]. Several studies have shown an inverse relationship between engagement and burnout, supporting the hypothesis that they are opposing experiences in sport [6,10]. Schaufeli et al. [11] suggested that burnout and engagement are opposites at the higher-order level, while exhaustion and vigor, as well as cynicism and dedication, are opposites at the dimension level. Furthermore, engaged athletes are more likely to experience other favorable experiences for their development, including positive affect [12], performance satisfaction [13], resilience [14], and flow [15].

Self-Determination Theory (SDT) is a core framework for understanding athlete engagement. It highlights the satisfaction of basic psychological needs as a key antecedent to optimal athlete functioning [15]. Therefore, being connected to the sporting environment (need for relatedness), making decisions and being involved in the activity (need for autonomy), and perceiving oneself as effective within the sporting context (need for competence) have a positive impact on engagement in young athletes across different competitive levels [16]. In this sense, self-determined motivation has been identified as a mediator in the aforementioned relationship [17,18].

According to SDT, an environment (e.g., coaches, parents) that supports athletes’ autonomy, competence, and relatedness leads to higher levels of athlete engagement [13,19]. A recent study suggested that a parent’s task-involving climate had a positive association with their athlete child’s engagement [20]. Furthermore, coaches who possess emotional-healing competency, which is defined as the ability to recognize and address the athletes’ emotional states and provide assistance, can effectively promote engagement among young athletes [21].

Lonsdale et al. [5] conducted a qualitative exploration of athletes experiences of engagement in New Zealand, identifying three initial components: confidence, vigor, and dedication. A second study, including athletes from New Zealand and Canada, confirmed this model and added enthusiasm as a fourth component. The final scale showed satisfactory psychometric properties, presenting a multidimensional structure with four factors [6].

Since the creation of the Athlete Engagement Questionnaire (AEQ), it has been translated, adapted, and validated in several countries, including Portugal [22,23], Croatia [24], Spain [10,25,26], Turkey [27], and China [28], and has also been used in athletes from Sweden [12]. The psychometric properties of the various AEQ versions are presented in Table 1.

These results provide support for the cross-cultural validity of the scale, confirming the original multidimensional structure and showing evidence for construct validity and internal consistency. Although most versions included young athletes in their samples, they did not assess the complete age range of youth or adapt the scale exclusively for this population. Similarly, studies have not considered the potential interaction between age, gender, and competitive level. A number of studies with Argentinean adolescents have shown the influence of gender, age, and competitive level on a range of variables linked to sport, including character strengths [29,30], passion, and life aspirations [31] and flow [32]. In this study, the competitive level was considered in accordance with Swann et al. [33]. Applying the equation to determine the ‘eliteness’ of the sample of athletes, the group with the highest competitive level emerged as ‘competitive elite’. This group is characterized by athletes competing at the highest level of their sports as representatives of their country in international tournaments, including Olympic sports, but who have not yet been successful at that level.

Therefore, the current study aims to examine athlete engagement and its relationship with indicators of the quality of the youth athlete’s sport experience, such as motivation, enjoyment, need satisfaction, and burnout. The study will explore differences according to gender, age, competitive level, and their respective interactions. Additionally, this study will confirm the factor structure of the AEQ in young athletes from Argentina, examine its validity and reliability, and test its measurement invariance. The study of engagement in sport and related variables can contribute to the promotion of contexts for the development of this type of experience, which is linked to positive youth development and health. In contrast to other countries, such as Denmark [34], in Argentina, the leading role is assumed by clubs as the primary setting for the initiation of sport and talent development, as opposed to schools [35], underscores the importance of focusing on these contexts of sport socialization for the promotion of physically active lifestyles among adolescents and emerging adults.

## 2. Materials and Methods

### 2.1. Participants

The study sample was composed of 1188 athletes aged between 10 and 26 years (*M* = 15.92; *SD* = 2.50) who participated in team (80.60%) and individual sports (19.40%). Gender was approached from a binary perspective, with participants self-identifying as either male (56.10%, *n* = 667) or female (43.90%, *n* = 521). Athletes belonged to the competitive regional level (73.5%, *n* = 873) within Buenos Aires (Argentina), and 26.5% (*n* = 315) had participated as a national athlete representing Argentina in international competitions at some point in their sporting career. The socio-demographic characteristics of all participants and comparison groups are presented in Table 2.

### 2.2. Measures

A custom-designed socio-demographic questionnaire was used to assess gender, age, sport, years of sport practice, hours of sport practice, and whether they had participated as athletes representing the national team of their sport and to specify when and in which tournaments or competitions they had participated.

The Athlete Engagement Questionnaire (AEQ) [6] was used to measure the four dimensions of engagement (confidence, vigor, dedication, and enthusiasm) through 16 items on a five-point Likert scale. To ensure translation accuracy, the AEQ was first translated from the English version into Argentinean Spanish by two researchers and an experienced Spanish sports psychologist, following the current recommendations of the International Test Commission [36]. Afterwards, to verify item equivalence, two native Argentinean English speakers conducted a back-translation into English. Finally, a bilingual expert was consulted to assess any differences in meaning between the original items and the back-translated items. This procedure confirmed that the version used in Spain [10] achieved linguistic equivalence.

The Spanish version [37] of the Behavioral Regulation in Sport Questionnaire (BRSQ-6) [38] was used to assess motivation in sport practice based on the SDT framework. The instrument comprises six subscales, each consisting of four items, and is designed to measure amotivation, external, introjected, identified, and integrated regulations, as well as intrinsic motivation, using a five-point Likert scale ranging from strongly agree to strongly disagree. Based on previous research [39,40], items from the subscales of intrinsic motivation, integrated regulation, and identified regulation were combined to create the variable “autonomous motivation”. Similarly, items from the subscales of introjected regulation and external regulation scales were combined to create the variable “controlled motivation”. Therefore, the scales of autonomous motivation (α = 0.82), controlled motivation (α = 0.84), and amotivation (α = 0.84) were used for this study.

To assess the degree to which athletes perceive satisfaction of their competence, autonomy, and relatedness needs, the Basic Psychological Needs Satisfaction Scale (BPNSS) [41] was used. The scale has 15 items grouped into three subscales. Responses were recorded on a five-point Likert scale of agreement. Previous studies demonstrated the psychometric properties of the scales used to measure basic need satisfaction among youth sport participants [42]. In this sample, the alpha coefficient was 0.84 for competence, 0.57 for autonomy, and 0.85 for relatedness.

The Sport Enjoyment Scale (SES) [43] was used to measure the experience of enjoyment in sports and its defining components through 17 items on a five-point Likert scale. The scale shows excellent psychometric properties for use with youth in Argentina, with an alpha coefficient of 0.82 for this sample.

The Spanish adaptation [44] of the Athlete Burnout Questionnaire (ABQ) [45] was used to assess the frequency of athletes’ perceptions of physical and emotional exhaustion, reduced sense of accomplishment, and devaluation of sport practice. The ABQ comprises nine items and five response options. The validity and reliability of the scale were confirmed in previous studies [44,45]. For this study, the total score of the scale was used (α = 0.72).

### 2.3. Procedure

The authorities of the sports clubs and institutions were contacted to invite them to participate in the study. The objectives and procedure to be followed were explained, and it was emphasized that the participation of athletes was anonymous and voluntary. Once authorization was obtained, contact was made with the coaches, who were responsible for training each of the groups of athletes. The day and time for data collection were coordinated with the coaches before or after training sessions.

For the participation of adolescents under 18 years of age, informed consent was requested from their parents or legal guardians. During the administration of the questionnaires, two researchers were always present to provide information on how to fill out the questionnaires and to resolve any doubts that might arise during the process. It was emphasized that participation was voluntary and anonymous, that they could withdraw from the study at any time without consequence, and that the data collected would be processed only by the researchers. In order to guarantee the players’ freedom and confidentiality when answering, coaches, club managers, and parents were not allowed to be present during the data collection. Although data collection was carried out in the groups formed by each of the teams, each athlete freely answered their own questionnaire protocol. The time required to fill out the scales was approximately 20 min, varying according to the age of the athlete.

In acknowledgement of the athletes’ participation and in accordance with the Psychodiagnostician’s Code of Ethics [46], which emphasizes the importance of communicating the results, reports on the general results were compiled and distributed to coaches in the majority of the teams or training groups, with the exception of those comprising a limited number of members. In all instances, the athletes who had participated could not be identified.

The present research adheres to local and international ethical and deontological standards and has been approved by the Responsible Conduct in Research Committee of the Faculty of Psychology at the University of Buenos Aires (Ref.: UBA-01.08.2017).

### 2.4. Data Analysis

Two variance component analyses were conducted using the least squares (VARCOMP Type I) and maximum likelihood (GLM) procedures to determine the normal distribution, linearity, and homoscedasticity of the sample [47,48]. The assignment variables were gender (g), competitive level (n), and age (e), and the outcome variable was scale scores (y).

Next, confirmatory factor analyses (CFA) were performed for the proposed models. The Unweighted Least Squares (ULS) method was selected because of the ordinal nature of the variables in this study [49]. Fit indices, including Chi-square, Comparative Fit Index (CFI), Tucker–Lewis Index (TLI), Root Mean Square Error of Approximation (RMSEA), and Standardized Root Mean Square Residual (SRMR), were calculated. Additionally, composite reliability, average variance extracted, convergent validity, and discriminant validity were estimated based on standardized residuals. Multigroup confirmatory factor analyses were conducted to test measurement invariance across gender, age periods (early adolescence—up to 15 years of age, late adolescence—between 15 and 19 years of age, and young adulthood—over 20 years of age), and competitive level (competitive elite vs. regional). Changes in model fit (ΔCFI ≤ 0.010, ΔTLI < 0.010, and ΔRMSEA < 0.015) were examined [50] at configural, metric, scalar, and strict levels of analysis.

Correlations with external measures such as motivation, satisfaction of basic psychological needs, enjoyment, and burnout were conducted. Multivariate analyses of variance (MANOVA) were performed to examine engagement and its factors with respect to gender, age, and competitive level. According to Cohen [51], a guideline for interpreting an eta square value (η2) is that 0.01 indicates a small effect, 0.06 indicates a moderate effect, and 0.14 indicates a large effect. Confirmatory analyses, measurement invariance, correlations, and analysis of variance were performed using R (v. 4.1.2), while SAS 9.2 was used for variance component analysis.

## 3. Results

### 3.1. Preliminary Analysis

The residual errors were found to be equal for the VARCOMP Type I and GLM procedures through variance component analysis. Therefore, it is assumed that the sample is linear, normal, and homoscedastic [48]. Table 3 shows the variance component analysis, descriptive statistics for the AEQ factors, and the internal consistency indices (Cronbach’s alpha).

### 3.2. Factorial Validity and Measurement Invariance

The four-factor model and second-order factor model (hierarchical model) showed optimal fit indices and error values (Table 4). Additionally, satisfactory validity and reliability indicators were found for the items and dimensions using the four-factor model (Table 5). All items had factor loadings above the recommended value of 0.30, the composite reliability values for the factors were at 0.80, and the average variance extracted values exceeded the suggested 0.50 [49]. In terms of the scale’s convergent validity, the *t* values were estimated. All values were significant (*t* ≥ 1.96), indicating that the scale items assess the same construct. Discriminant validity was established by calculating the average variance extracted for each latent variable [49].

Before testing measurement invariance, CFAs were conducted for both gender, age, and competitive level based on the four-factor model. Satisfactory fit indicators were found for each group (Table 6). Considering that test of measurement invariance is a critical prerequisite to comparing scores across groups [52], we carried out multigroup CFA across gender, age, and competitive level groups, testing several types of factorial invariance (configural, metric, scalar, and strict invariance). The study found robust measurement invariance across gender, age, and competitive level. This was supported by the fit indices, which did not significantly decrease despite the addition of constraints (Table 6).

### 3.3. Intrascale Correlations and Evidence of Validity Based on Relationships with Other Variables

Intrascale associations show moderate to high correlations among the factors of engagement and between them and the total score (Table 7). Likewise, moderate to strong direct correlations were found with autonomous motivation and enjoyment, and inverse correlations with amotivation and burnout. The relationships with controlled motivation were low, and there was no significant correlation with confidence. Regarding the perception of satisfaction of basic psychological needs, low to moderate correlations were found with autonomy and relatedness, while moderate to high correlations were found with the need for competence, particularly with the confidence factor.

The multivariate results showed that gender (Pillai’s trace = 0.01, *F*(4, 1101) = 5.19, *p* < 0.001, η2 = 0.02) and competitive level (Pillai’s trace = 0.02, *F*(4, 1101) = 5.19, *p* < 0.001, η2 = 0.02) have main effects. Additionally, there are interactions between gender and competitive level (Pillai’s trace = 0.01, *F*(4, 1101) = 2.48, *p* = 0.042, η2 = 0.01), and between age and competitive level (Pillai’s trace = 0.01, *F*(8, 2204) = 1.95, *p* = 0.048, η2 = 0.01). No main effect was found for age (Pillai’s trace = 0.01, *F*(8, 2204) = 1.60, *p* = 0.118, η2 = 0.01), or between gender and age (Pillai’s trace = 0.01, *F*(8, 2204) = 1.91, *p* = 0.053, η2 = 0.01). Similarly, there was no significant interaction between all three variables (Pillai’s trace = 0.01, *F*(8, 2204) = 1.48, *p* = 0.156, η2 = 0.01). Univariate analyses of the main effect revealed significant results for confidence with respect to gender and competitive level, as well as for vigor, and dedication with respect to age (Table 8).

Post hoc tests were calculated to determine if there were differences in the means of reported variables across the three age groups. The results showed that athletes aged up to 15 years had significantly higher levels of all engagement factors compared to athletes aged over 20 years (*p* = 0.009 confidence; *p* = 0.046 vigor; *p* = 0.001 dedication; *p* = 0.015 enthusiasm). Additionally, athletes aged up to 15 years showed significantly higher levels of dedication compared to all age groups (*p* = 0.007).

**Figure 1:**
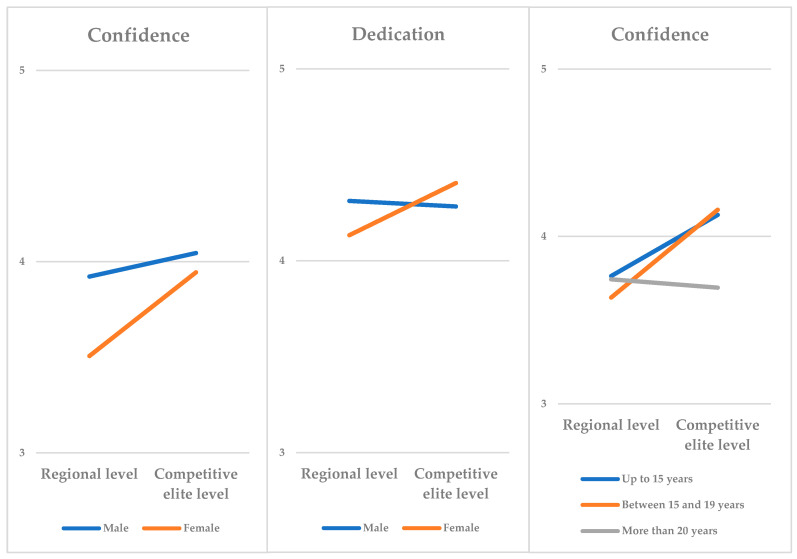
Interactions of gender, age, and competitive level on Athlete Engagement Questionnaire factors.

In terms of the interactions between factors, the study found that confidence and dedication varied in the interaction between gender and competitive levels (Figure 1). Males who participated as national athletes (competitive elite level) had the highest levels of confidence, while female athletes in lower competitive levels had the lowest levels. Therefore, the difference in confidence between genders was smaller (although still in favor of males) when the competitive level was high (*p* = 0.039). On the other hand, it was found that females in competitive elite level exhibited the highest levels of dedication, while female athletes in lower competitive levels had the lowest scores. In other words, dedication was greater among females at a higher competitive level (*p* = 0.027).

Furthermore, a significant interaction between age and competitive level was found for the confidence factor (Figure 1). Confidence remained high at the highest competitive level (elite level) until the age of 19, but decreased in the group of individuals over 20 years old. In contrast, in the lower competitive level group, confidence generally remained stable and showed a higher mean in the older age group (*p* = 0.015).

Finally, a significant interaction was found between gender, age, and competitive level (*p* = 0.022). It was observed that males who participated as national athletes (competitive elite) consistently displayed higher confidence levels, except for individuals over 20 years old, where the difference was reversed. Among females, those at higher competitive levels consistently displayed higher confidence levels, although the extent of the differences varied according to age.

## 4. Discussion

The aim of this study was to examine athlete engagement and its relationships with indicators of the quality of the athlete’s sport experience while exploring potential gender, age, and competitive level differences. Furthermore, this study validated the Athlete Engagement Questionnaire (AEQ) in young athletes and confirmed its factor structure. To achieve this, the factorial structure of the scale using confirmatory factor analysis (CFA) for diverse models was first tested. The results provided support for the hypothesized four-factor and second-order (hierarchical) structures. Additionally, item factor loadings, composite reliability, average variance extracted, and convergent and discriminant validity of the engagement dimensions were found to be satisfactory, indicating good psychometric properties of the AEQ. Reliability analyses were also conducted, resulting in satisfactory Cronbach’s alpha coefficients. These findings are consistent with previous research [10,22,23,24,25,26] that has examined various versions of the scale, confirming the robust functioning of the construct within the sports context.

Moreover, measurement invariance analysis revealed satisfactory performance of the questionnaire across gender, age, and competitive level. These findings are aligned with previous research [10] and provide valuable insights into the psychometric properties of the AEQ for young athletes of different genders and competitive levels. These findings can be beneficial for future follow-up studies and facilitate the transition from lower to higher competitive levels, as the questionnaire demonstrated consistent measurement across all groups.

Consistent with previous research in other contexts [6,22,24,25,27], the findings of the present study confirm moderate to strong correlations among the engagement dimensions. This supports the notion that engagement is a multidimensional construct consisting of various variables. Additionally, a moderate to high negative association between engagement and burnout supports the notion that these constructs are opposites [11], as also shown in previous studies [10,53].

The results showed additional validity evidence of the AEQ by the expected correlations between engagement and motivation, enjoyment, and the satisfaction of the basic psychological needs (i.e., autonomy, competence, and relatedness). However, no studies have been found that relate these variables. It is noteworthy that the correlations with the motivational variables revealed differences in the dimensions of engagement. In particular, the confidence variable exhibited the lowest correlations with the motivational regulations (even a non-significant relationship with controlled motivation) and the strongest relationships with the need for competence and the need for autonomy. This could be explained by its more cognitive component, as opposed to the other variables, which are defined more by their emotional component, oriented to the perception of competence and not so much to the enjoyment and energy put into the activity. Therefore, this study provides new evidence of validity, specifically in the Argentinean context, regarding the relationship between engagement and other variables associated with performance and well-being in sports [16,17,18].

While previous validations have demonstrated that the model is invariant by gender [23,28] and across different competitive levels [25], the effects of athletes’ age and its interactions on engagement have not been examined in previous literature. Therefore, multivariate analyses were conducted, revealing that male athletes reported significantly higher levels of confidence compared to female athletes. This finding is consistent with previous studies conducted with Portuguese athletes [14,23]. However, it is worth noting that gender differences in confidence were not found among sprinter runners from Croatia in the Babić et al. study [24], although the mentioned study involved a very specific sample of 71 elite athletes. Several studies addressing the role of psychological skills for performance across various competitive levels consistently highlight gender differences in favor of males regarding self-confidence [54,55]. However, these studies have not considered the potential interaction between gender and competitive level. In the present study, it was found that male athletes perceive higher levels of confidence at regional competitive levels than at elite levels. This suggests that gender differences are equalized at the elite level, consistent with the results shown by Babić et al. [24].

Additionally, the present study reveals an interesting finding about dedication levels, with women at the competitive elite level achieving the highest scores and men from the regional level obtaining the lowest scores. Gender and competitive level may impact athletes’ engagement in different ways. The efficacy belief component of engagement, which refers to confidence, and the desire to invest effort and time in the sport, known as dedication, are affected differently by gender and competitive level. Diverse theoretical models [56] show that gender stereotypes may affect athletes’ self-perception of competence and the importance they attach to their sport practice, which in turn impacts their sport participation and performance. In this line, it was found that among high-level competitive athletes, gender differences disappear when comparing their perception of efficacy, showing that in contexts with strong gender stereotypes, such as the competitive sport environment, it is possible not to find these differences because role expectations are the same for males and females [57].

Regarding age, athletes up to 15 years old reported the highest levels of all engagement dimensions compared to those over 20 years of age and showed significantly higher levels of dedication compared to all other age groups. This age effect has been observed in previous studies [14,58], which have shown that athletes under 18 exhibit higher levels of vigor and enthusiasm compared to older athletes. As athletes age, they begin to acquire psychological skills related to sports performance, such as self-confidence and/or concentration skills [58]. However, sports practice can also increase expectations and demands for improvement and results, which can lead athletes to be more critical of their own performance [14] and feel greater pressure from their environment. This increased pressure may affect the dimensions of engagement that relate to enjoyment and effort during practice, such as enthusiasm, dedication, and vigor.

In Argentina, as in most countries worldwide, young people finish or have already finished secondary school around the age of 17. This event has a significant impact on the lives of adolescents. In general, during this transition period towards emerging adulthood, young people must decide how they will continue their lives beyond high school, progressively acquiring a greater number of responsibilities and roles [59,60]. In many cases, academic and work demands also increase, resulting in a reduction of time available for recreational activities such as sports and leisure [60]. This may explain the observed decrease in engagement with sports practice as age increases.

Athletes at the competitive elite level exhibit higher confidence scores than those at the regional level. This finding is in line with Babić et al. [24], who found that athletes in this group perceived more enthusiasm. Additionally, Kristensen [61] found that athletes who trained for more than 19 h per week reported higher levels of engagement. This criterion is based on the amount of dedication and can be used to characterize elite-level athletes who typically dedicate many more hours than those at lower competitive levels. However, other studies have not found such differences. It is important to consider participants’ characteristics. For example, Sindik and Čuk [58] had a homogeneous sample of male handball players. However, Pedro and Veloso [14] did not find any differences based on the competitive level. It is important to note that their sample had a wide age range (12 to 31 years) and found differences based on age, although they did not assess its interaction. Furthermore, the definition of elite athletes in sport psychology research has been inconsistent and confusing, which can have an impact on studies conducted in this area [33]. Nevertheless, studies conducted on Spanish taekwondo and wrestling athletes [54,62], as well as on various athletes in Argentina [55], indicate that individuals who compete at a higher level tend to perceive a greater need for psychological skills to enhance their performance compared to those who participate at a regional or recreational level.

The present study found an interesting correlation between age and competitive level, particularly in relation to confidence. Athletes at the highest competitive level (competitive elite) maintain consistently high levels of confidence until the age of 19, but confidence decreases among those over 20 years old. Conversely, confidence tends to remain stable in the lower competitive level group, with the older age group exhibiting a higher average level of confidence. The assessment of confidence, as a part of the engagement, reflects the perception of competence, which is crucial for athletic performance and satisfaction with practice [5]. It is worth noting that precisely from the age of 20 onwards, when having a strong sense of confidence becomes even more important, as it generally coincides with the highest level of athletic development (i.e., participating in a senior national team), athletes reported lower levels of confidence compared to others. Further investigation is necessary, considering environmental factors such as the athlete–coach relationship [63]. This could explain the decline in perceived competence among these athletes, which ultimately affects their performance and positive engagement experience. Research has shown that the perceived motivational climate among basketball and volleyball players becomes more controlling and ego-oriented in the under-19 category compared to the under-15 and under-17 categories [64]. This negative impact on the quality of the sports experience has been documented [65].

Finally, the present study found that male athletes in the competitive elite level category showed higher levels of confidence, dedication, and enthusiasm compared to other male athletes, except for those aged over 20, where the difference favored younger athletes. Among female athletes, those at higher competitive levels consistently showed higher levels of confidence, dedication, and enthusiasm, with some variations depending on age. In addition to the previous literature, it is important to highlight that the lower perception of engagement dimensions in older athletes may also be linked to the greater development and professionalization of male sports. In Argentina, football, basketball, and tennis are the most popular sports with higher exposure and professional development structures, primarily in their male divisions [66]. This can also contribute to increased pressure at the highest competitive level (i.e., national teams level), which could affect athletes’ confidence, dedication, and enthusiasm for the sport.

The present study is not exempt from limitations. The sample mostly consisted of team sports athletes (80.6%), which limits exploration of differences between team and individual sports, as seen in previous studies [14,20,53]. As stated by Storm et al. [34], differences between sports regarding athletes’ opportunities to progress from regional level to national representation (elite level) may be due to the prevalence and spread of their sport. Argentina is a country with a large passion for team sports, with disciplines such as football, basketball, field hockey, rugby, and volleyball demonstrating the greatest development and organizational structure. Throughout history, Argentina has achieved notable success at the international level, including at the Olympic Games, where it has competed on an equal footing with countries with more extensive sporting infrastructures. It is also important to consider the socio-economic disparities that exist within a country as diverse as Argentina. A study showed that adolescent Olympians (preparing for the 2018 Youth Olympic Games in Buenos Aires) were more likely to come from households with higher educational attainment and more socially advantaged backgrounds compared to other groups of adolescents [67]. Conversely, there is a lack of uniformity in sports development across the country, with the majority of athletes concentrated in the metropolitan area of Buenos Aires (the capital of the country). The country’s sole National High Athletic Performance Center (CeNARD) is responsible for training all sports disciplines representing the nation, with the exception of football, which has its own training center with dedicated facilities in Buenos Aires. In the future, it would be beneficial to consider these socio-economic and cultural factors in order to gain a deeper understanding of the career development of elite athletes [34]. Moreover, the present study focused on the highest competitive level, examining those athletes who participated as national athletes representing Argentina in their sport but were not exclusively training with the National Team at the time of data collection. It would be interesting to investigate further how engagement may vary according to these situations.

Additionally, the cross-sectional design does not allow for follow-up engagement over time. Longitudinal studies have measured engagement over a season, examining antecedents such as variables in the environment, including the athlete–coach relationship [19]. Follow-up studies of athletes’ careers could provide information about the predictive validity of the construct. Future research directions may include studying the protective effect of engagement in relation to other experiences of sports distress such as pressure, anxiety, and dropout. Additionally, exploring engagement in other contexts, such as physical education and exercise settings, as well as examining other favorable indicators of sports participation (e.g., adherence, enjoyment in practice), and health and risk behaviors (e.g., substance use) [68], could be beneficial. Finally, exploring the engagement of coaches and its impact on the experience of youth athletes, as well as strategies to prevent burnout [7], can provide a pathway for interventions involving these socially significant agents in the sports domain.

## 5. Conclusions and Practical Implications

In summary, engagement is a crucial factor in improving athletes’ motivation and positively influencing their personal and social functioning. Reliable assessment instruments, adapted to specific contexts, provide sport psychologists with robust tools to support positive youth development through sport. These findings also support a multidimensional approach to engagement. Moreover, this study supports the role of engagement as the opposite of burnout and highlights its correlation with positive practice experiences.

This study also provides coaches, parents, and sports leaders with valuable knowledge and resources to enhance positive sporting experiences and promote health. Following several studies that have shown the significance of the creation of motivational climates by coaches and their impact on engagement, particularly in what happens during competitions and matches [19], it opens the way for intervention, providing specific strategies to develop in these contexts to support autonomy and promote task-involving climates. For instance, coaches can set challenging and intrinsically motivating goals and establish a line of learning for each athlete to evaluate their progress. With respect to parents, as key figures for athletes in the initial contact with sport, the provision of workshops to reflect on effective practices to accompany, provide instrumental support, and act as a buffer when difficulties arise can contribute to the development of engagement in their sons and daughters.

The role of sport stakeholders, such as federation leaders, is to create opportunities for young athletes that allow them to choose sport as a possible path, among others. To this end, support and accompaniment for dual careers, providing economic and structural support for normative transitions (i.e., moving from school to university) and non-normative transitions (i.e., moving, injuries, etc.), can be specific actions to accompany athletes from adolescence to emerging adulthood, promoting positive development trajectories, and avoiding the decrease in sport engagement that was found in this study from the age of 19. Likewise, they are responsible for providing training to coaches and parents.

Finally, to continue narrowing the gender gap that persists, it will be important to further promote women in leadership positions, such as coaching and management. Women in these positions can influence cultural change by challenging gender stereotypes and demonstrating that sport can be a desirable and expected context for girls and women and that equity is achievable [69]. Despite the aforementioned benefits, the prevalence of women in coaching roles within the sports domain remains low [70]. This may have an adverse effect on the engagement of women in sporting activities.

## Figures and Tables

**Table 1:** Psychometric properties of Athlete Engagement Questionnaire versions and validations.

	Sample		Psychometric Properties		
Authors	Country	Population	Validity		Reliability
			Factorization	Other Types	
Lonsdale, Hodge, and Jackson [6]	New Zealand and Canada	Study 1: 729 athletes from different sports, Study 2: 201 elite athletes (*M* age = 22.9, *SD* = 7.20), Study 3: 343 athletes (*M* age = 24.5, *SD* = 7.70)	Principal Axis Factor analysis. Confirmatory Factor Analysis (CFA): four first-order factors and second-order engagement construct	- Content validity (expert judges and pilot study) - Criterion related validity (burnout)	Cronbach’s Alpha (between 0.84 and 0.89)
Martins et al. [22,23]	Portugal	771 athletes (*M* age = 16.9, *SD* = 4.51) from different competitive levels (i.e., elite, national, and regional competitive levels)	CFA: four-factor model	- Content validity (expert judges) - Cross validity (Multigroup analysis)	Composite reliability (between 0.85 to 0.88)
Babić et al. [24]	Croatia	71 top athlete sprinters (participation in sports between 12 and 13 years)	Principal Axis Factoring (PAF): four-factor model	- Criterion-related validity (athletic identity)	Cronbach’s Alpha (between 0.76 and 0.88)
De Francisco et al. [10,25,26]	Spain	Study 1: 509 athletes (*M* age = 17.36, *SD* = 4.58) Study 2: 1157 athletes (*M* age = 17.55, *SD* = 4.25)	CFA: one, four, and a second-order (hierarchical) factor model	- Content validity (focus group) - Measurement model invariance across genders and competitive levels - Criterion-related validity (burnout)	Cronbach’s Alpha (between 0.80 and 0.85)
Kelecek et al. [27]	Turkey	201 athletes (*M* age = 23.3, *SD* = 2.84)	Principal Component Analysis (PCA): four-factor model	- Criterion-related validity (burnout)	Cronbach’s Alpha (between 0.75 and 0.92)
Jiang et al. [28]	China	379 athletes (*M* age = 16.07, *SD* = 1.15)	CFA: four-factor and second-order models.	- Content validity (expert judges and pilot study)	Composite reliability (between 0.88 and 0.90)

**Table 2:** Socio-demographic characteristics of participants.

	Total Sample	Competitive Elite Level	Regional Level
Gender	667 (56.1)	164 (52.1)	503 (57.6)
Age	15.92 (2.50)	15.21 (2.12)	16.18 (2.58)
Practicing days per week	3.79 (1.45)	4.73 (1.58)	3.33 (1.12)
Practicing hours per session	2.69 (1.21)	3.28 (1.47)	2.4 (0.93)
Sports			
Team	958 (80.6)	120 (38.1)	838 (96.0)
Volleyball	312 (26.3)	26 (8.3)	286 (32.8)
Basketball	284 (23.9)	35 (11.1)	249 (28.5)
Handball	113 (9.5)	43 (13.7)	70 (8.0)
Field hockey	97 (8.2)	5 (1.6)	92 (10.5)
Rugby	58 (4.9)	-	58 (6.6)
Football	56 (4.7)	6 (1.9)	50 (5.7)
Futsal	37 (3.1)	5 (1.6)	32 (3.7)
Cestoball	1 (0.1)	-	1 (0.1)
Individual	230 (19.4)	195 (61.9)	35 (4.0)
Swimming	33 (2.8)	18 (5.7)	15 (1.7)
Gymnastics	29 (2.5)	28 (8.8)	1 (0.1)
Yachting	24 (2.0)	24 (7.6)	-
Canoeing	18 (1.5)	18 (5.7)	-
Triathlon	13 (1.1)	13 (4.1)	-
Taekwon-do	12 (1.1)	11 (3.5)	1 (0.1)
Shooting	12 (1.1)	12 (3.8)	-
Figure skating	11 (0.9)	1 (0.3)	10 (1.1)
Others	78 (33.9)	70 (22.2)	8 (0.9)

Note: Gender is expressed in males n (%), sports are expressed in n (%). Age, days and hours of practice are expressed in means (SD). Other sports were equestrian, tennis, boxing, golf, cycling, judo, fencing, pentathlon, archery, table tennis, windsurfing, athletics, rowing, etc.

**Table 3:** Descriptive statistics of the Athlete Engagement Questionnaire factors for the total sample.

Variables	Mean (*SD*)	Min.–Max.	Skewness	Kurtosis	α	Residual Errors	
						VARCOMP	GLM
Confidence	3.81 (0.76)	1–5	−0.49	−0.12	0.81	52,198.75	52,198.75
Vigor	4.16 (0.62)	1–5	−0.50	−0.17	0.77	3404.37	3404.37
Dedication	4.30 (0.65)	1–5	−1.08	−1.23	0.81	3902.33	3902.33
Enthusiasm	4.58 (0.52)	1–5	−1.50	3.16	0.82	9914.18	9914.18
Engagement (total)	4.21 (0.51)	1–5	−0.69	0.60	0.90	20,405.00	20,404.57

Note: *SD* = standard deviation, α = Cronbach’s alpha, VARCOMP = least squares procedure; GLM = maximum likelihood procedure.

**Table 4:** Summary of model fit indices for all tested models of the Athlete Engagement Questionnaire.

Model	χ^2^ (*df*)	CFI	TLI	SRMR	RMSEA	CI_90%_ for RMSEA
One-factor	646.14 (104)	0.94	0.93	0.09	0.07	[0.06; 0.07]
Four-factor	161.01 (98)	0.99	0.99	0.05	0.02	[0.01; 0.03]
Second-order (hierarchical)	200.38 (100)	0.98	0.98	0.05	0.03	[0.02; 0.03]

Note: χ^2^ = chi square, *df* = degrees of freedom, CFI = Comparative Fit Index, TLI = Tucker–Lewis Index, RMSEA = Root Mean Square Error of Approximation, SRMR = Standardized Root Mean Square Residual, CI = confidence interval.

**Table 5:** Reliability and validity indices for the Athlete Engagement Questionnaire items.

Factor	Item	Loadings	Composite Reliability	AVE	Convergent Validity ^a^	Discriminant Validity
Confidence	1	0.75	0.84	0.58	30.16	0.32 < 0.54; 0.48 < 0.62; 0.23 < 0.69
	5	0.86			32.12	
	9	0.65			27.13	
	13	0.77			30.16	
Vigor	2	0.76	0.80	0.54	32.69	0.58 < 0.62; 0.71 > 0.69
	6	0.74			32.29	
	10	0.81			34.49	
	14	0.63			28.30	
Dedication	3	0.81	0.88	0.62	35.96	0.61 < 0.69
	7	0.80			35.56	
	11	0.74			33.66	
	15	0.80			35.39	
Enthusiasm	4	0.87	0.92	0.69	38.50	-
	8	0.88			38.80	
	12	0.84			37.21	
	16	0.71			33.26	

Note: AVE = average variance extracted; ^a^ *t* values.

**Table 6:** Measurement invariance of the Athlete Engagement Questionnaire for gender, age, and competitive level.

Model	χ^2^ (*df*)	CFI	TLI	SRMR	RMSEA	CI_90%_ for RMSEA	ΔCFI	ΔTLI	ΔRMSEA
*Gender*									
Males	78.55 (98)	1.00	1.00	0.05	0.00	[0.00, 0.01]			
Females	105.80 (98)	0.99	0.99	0.06	0.01	[0.00, 0.03]			
Configural invariance	184.35 (196)	1.00	1.00		0.00		-	-	-
Metric invariance	220.140 (208)	0.99	0.99		0.01		−0.012	−0.002	0.010
Scalar invariance	228.63 (220)	0.99	0.99		0.01		0.001	0.001	−0.002
Strict invariance	257.63 (236)	0.99	0.99		0.01		−0.002	−0.002	0.004
*Age*									
Up to 15 years old	61.12 (98)	1.00	1.00	0.06	0.01	[0.00, 0.00]			
Between 15 and 19	106.77 (98)	0.99	0.99	0.05	0.01	[0.00, 0.02]			
Over 20	24.92 (98)	1.00	1.22	0.08	0.00	[0.00, 0.00]			
Configural invariance	192.82 (294)	1.00	1.00		0.00		-	-	-
Metric invariance	220.163 (318)	1.00	1.00		0.00		0.000	0.000	0.000
Scalar invariance	227.92 (342)	1.00	1.00		0.00		0.000	0.000	0.000
Strict invariance	252.97 (374)	1.00	1.00		0.00		0.000	0.000	0.000
*Competitive level*									
Competitive elite level	45.96 (98)	1.00	1.04	0.06	0.00	[0.00, 0.00]			
Regional level	117.92 (98)	0.99	0.99	0.05	0.01	[0.00, 0.02]			
Configural invariance	163.88 (196)	1.00	1.00		0.00		-	-	-
Metric invariance	173.32 (208)	1.00	1.00		0.00		0.000	0.000	0.000
Scalar invariance	186.77 (220)	1.00	1.00		0.00		0.000	0.000	0.000
Strict invariance	202.53 (236)	1.00	1.00		0.00		0.000	0.000	0.000

*Note:* χ^2^ = chi square, *df* = degrees of freedom, CFI = Comparative Fit Index, TLI = Tucker–Lewis Index, RMSEA = Root Mean Square Error of Approximation, Δ = fit index variation.

**Table 7:** Correlations between the Athlete Engagement Questionnaire factors and motivational regulations, enjoyment, burnout, and psychological needs satisfaction.

Variables	Confidence	Vigor	Dedication	Enthusiasm	Engagement (Total)
Confidence	-				
Vigor	0.42 **	-			
Dedication	0.53 **	0.58 **	-		
Enthusiasm	0.35 **	0.62 **	0.60 **	-	
Engagement (total)	0.76 **	0.81 **	0.85 **	0.77 **	-
Autonomous motivation	0.27 **	0.40 **	0.41 **	0.33 **	0.45 **
Controlled motivation	−0.08	−0.21 **	−0.14 **	−0.29 **	−0.22 **
Amotivation	−0.19 **	−0.36 **	−0.34 **	−0.46 **	−0.42 **
Enjoyment	0.39 **	0.54 **	0.50 **	0.46 **	0.58 **
Burnout	−0.42 **	−0.37 **	−0.45 **	−0.32 **	−0.50 **
Autonomy satisfaction	0.28 **	0.27 **	0.19 **	0.15 **	0.28 **
Relatedness satisfaction	0.18 **	0.27 **	0.19 **	0.22 **	0.26 **
Competence satisfaction	0.69 **	0.45 **	0.43 **	0.37 **	0.60 **

** *p* < 0.01.

**Table 8:** Univariate analyses of the Athlete Engagement Questionnaire factors for gender, age, and competitive level.

**Gender**					
	**Male** **Mean (*SD*)**	**Female** **Mean (*SD*)**		***F***	**η2**
Confidence	3.94 (0.70)	3.63 (0.79)		11.55 **	0.01
Vigor	4.21 (0.61)	4.07 (0.62)		3.36	0.00
Dedication	4.35 (0.63)	4.23 (0.66)		0.17	0.00
Enthusiasm	4.60 (0.51)	4.55 (0.51)		0.51	0.00
**Age**					
	**Up to 15** **Mean (*SD*)**	**Between 15 and 19** **Mean (*SD*)**	**Over 20** **Mean (*SD*)**	***F***	**η2**
Confidence	3.88 (0.69)	3.79 (0.79)	3.65 (0.73)	2.20	0.00
Vigor	4.18 (0.62)	4.16 (0.63)	4.02 (0.51)	3.62 *	0.01
Dedication	4.35 (0.60)	4.30 (0.67)	4.09 (0.58)	3.94 *	0.01
Enthusiasm	4.62 (0.49)	4.58 (0.53)	4.47 (0.44)	2.44	0.00
**Competitive Level**					
	**Competitive Elite Level** **Mean (*SD*)**	**Regional** **Level** **Mean (*SD*)**		***F***	**η2**
Confidence	4.13 (0.62)	3.70 (0.77)		13.58 ***	0.01
Vigor	4.20 (0.61)	4.14 (0.62)		0.31	0.00
Dedication	4.46 (0.57)	4.24 (0.66)		3.18	0.00
Enthusiasm	4.60 (0.52)	4.57 (0.50)		0.01	0.00

Note: *SD* = standard deviation, η2 = eta square (effect size). Post hoc tests for age are explained in the text. * *p* < 0.05; ** *p* < 0.01; *** *p* < 0.001.

## Data Availability

The data presented in this study are openly available and shared at: http://hdl.handle.net/11336/182530, accessed on 3 January 2024.
